# An empirical study on the teaching mode of cultural translation in college English based on the Production Oriented Approach (POA)

**DOI:** 10.1371/journal.pone.0326127

**Published:** 2025-06-27

**Authors:** Tong He, Chongyue Li

**Affiliations:** 1 Jingjiang College, Jiangsu University, Zhenjiang, P. R. China; 2 School of Foreign Languages, Jiangsu University, Zhenjiang, P. R. China; Ahvaz Jundishapur University: Ahvaz Jondishapour University of Medical Sciences, IRAN, ISLAMIC REPUBLIC OF

## Abstract

Under the circumstances of “Curriculum ideological and political”, in order to adapt to the reform of college English teaching and the needs of Education for Sustainable Development (ESD), as well as optimize the A-level distinguishable teaching mode of college English stratified teaching, this paper takes the fifth stage of “Production Oriented Approach” (POA) as the theoretical basis and completes a one-semester mixed teaching experiment for the Chinese culture translation section in *New Horizon College English*. Taking the paragraph translation of “Tai Chi” as an example, a detailed teaching design and implementation process were presented, attempting to construct a sustainable blended teaching model for the cultural translation section of college English. Quantitative and qualitative data statistics and analysis were used to evaluate and reflect on the effectiveness of teaching practice. The research results indicate that compared to traditional college English teaching, blended teaching model based on POA can enhance the English language and cultural output ability of A-level students. The mode is of sustainable value, which is beneficial to students understanding of Chinese culture, thereby enhancing their cultural confidence. In addition, this model can effectively enhance teachers’ ability to apply theoretical knowledge in blended teaching design, which has certain significance for the college English curriculum reform and the practice of “curriculum-based political and ideological education”.

## 1. Introduction

Under the background of deepening undergraduate education reform and improving teaching quality for sustainable development of education, the *Guidelines for College English Teaching* (2020 edition) explicitly states: “College English teaching can cultivate students’ understanding and interpretation abilities of Chinese culture, and serve the external dissemination of Chinese culture” [1–3]. The *Guide* also emphasizes: “By learning and using English, college students can enhance the country’s language strength, effectively spread Chinese culture, promote extensive exchanges with people from various countries, and enhance the country’s soft power” [4–6]. However, the cultural dissemination ability of college students largely depends on their cultural translation ability [7,8]. Therefore, the teaching of Chinese cultural translation in college English has a long way to go [9–11].

**Fig 1:**
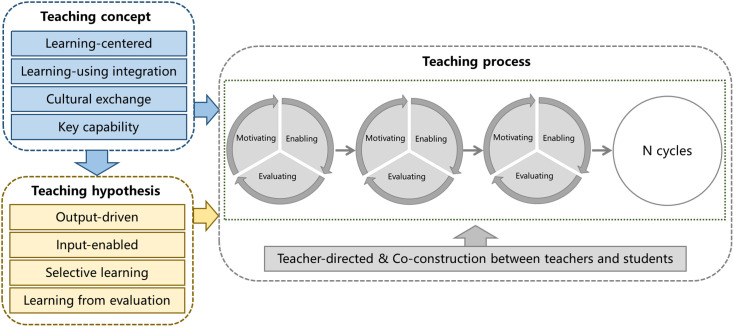
The fifth stage of POA theoretical system.

In order to solve the long-standing problem of “separation in learning and application” in Chinese foreign language teaching and adapt to the sustainable development of education, Professor Wen Qiufang and her group proposed the “Production Oriented Approach” (POA) for college foreign language classroom-teaching in 2015 [12–14]. This theoretical system consists of three parts: teaching concept, teaching assumptions, and teaching processes [15]. After ten years of continuous development and improvement, it has now been revised to the fifth stage, as shown in Fig 1. The specific changes in the revised POA theoretical system mainly include the addition of “cultural exchange theory” in the teaching concept section, and the replacement of “whole person education theory” with “key capability theory” [16]. The teaching process part makes the three stages of motivating, enabling, and evaluating into internal small cycles, and the small cycle will form a whole large cycle through N times [17]. The theory simultaneously emphasizes the combination of “teachers’ guiding role” and “teacher-student co-construction” in the teaching process [16,18]. Among them, the “cultural exchange theory” aims to guide the correct handling of the relationship between the target language culture and the learner’s mother tongue culture in teaching Chinese as a foreign language, emphasizing the integration of Chinese and foreign cultural learning into language teaching, and fully utilizing the role of language as a cultural carrier [19,20]. The “cultural exchange theory” is also applicable to guiding Chinese college students in cross-cultural input and output activities in English classrooms [21]. Classroom teaching adopts the strategy of “explicit language and implicit culture” to ensure students to enhance cultural literacy in the process of language acquisition [22,23]. What’s more, contemporary college students will be able to fairly and objectively understand and respect the culture of English-speaking countries and local Chinese culture.

It is pointed out during the formation of POA by Professor Wen Qiufang that the classroom teaching theory is mainly applicable to middle and advanced foreign language learners [24]. The college where the author works carried out the reform of hierarchical teaching of college English since 2018. The reform mainly adopts educational methods that balance differential development with distinctive features to solve the problem of uneven development in college English learning, and to maximize the improvement of students’ English proficiency at all levels [25]. Although A-level students entering the advanced stage are well in English language foundation and language skills (with average score above 115 in the college entrance examination), they encounter a common output dilemma when dealing with the Chinese English translation section of the textbook (which involves topics such as China’s history, culture, economy, and social development) in the classroom. This is called “cultural aphasia”, which highlights the separation between language proficiency and cultural quality in core competencies [21,26]. Therefore, the POA is more suitable for the sustainable development teaching practice of the A-level cultural translation section in college English hierarchical teaching, in order to promote the improvement of students’ cultural output abilities.

## 2. Materials and methods

### 2.1. Research questions

The college where the author works does not specifically set up “Chinese Culture” English general education courses for non-English major students. Therefore, cultural themes in college English have to be taught through the cultural translation section of the current textbook *New Horizon College English*. This teaching experiment aims to solve three main problems:

(1) Can POA raise students’ interest and confidence in translation studying?
(2) Compared with traditional college English translation teaching, can POA improve students’ understanding abilities of Chinese culture?
(3) Can POA enhance cultural translation abilities of students in English learning?

### 2.2. Research subjects

To clarify the issues above, the author conducted a one-semester teaching experiment in the first semester of the freshman year, taking two liberal arts classes in accounting major of grade 2022 in the college as the research subjects. Students in accounting major have a relatively good foundation in English, and most of them can reach the A-level standards. But some of them lack learning initiative and have no clear learning objectives. These two classes are named as an “experimental group” and a “control group”, respectively [27]. The control group (n = 31) adopted the traditional process: before class, students complete cultural translation themselves—in class, the teacher explains the translation answer sentence by sentence—after class, the teacher evaluates and summarize, while the experimental group (n = 31) adopted POA as teaching method for Chinese cultural translation section of *New Horizon College English* (Ideological and Political Wisdom Edition) [28]. Considering that freshmen have just entered the university and their English scores in the college entrance examination are somewhat convincing, SPSS 26.0 was used to conduct statistical analysis on these students, and the results are shown in Table 1 [29,30].

**Table 1:** Comparison of English scores in the college entrance examination between the control group and the experimental group.

Group	Numbers of cased	Lowest score	Highest score	Mean	Standard deviation	t value	p value
Control group	31	88	131	116.32	9.253	0.509	0.613
Experimental group	31	92	129	115.13	9.211		

From Table 1, it can be seen that the average score of the control group in the college entrance examination was 116.32 points (SD = 9.253), while the average score of the experimental group in the college entrance examination was 115.13 points (SD = 9.211), with no significant inter group difference (t = 0.509, P = 0.613 > 0.05), indicating that there was no significant difference in English proficiency between the two groups of students before the experiment [29].

### 2.3. Experimental procedure

**Fig 2:**
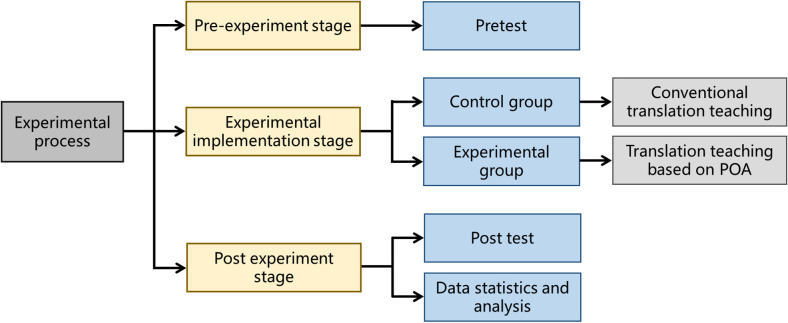
Teaching experiment procedure.

This semester the teacher arranged five units in the first volume of *New Horizon College English* (Ideological and Political Wisdom Edition) and conducted five cultural translation teaching practices. The teaching experiment procedure was shown in Fig 2 [31]. Due to time and space limitations, this section takes the after-school translation exercise of Unit 4— “Tai Chi” as an example to demonstrate how to follow the teaching process of POA for blended teaching of online and offline Chinese cultural paragraph translation, how to make small output goals serve large output goals, and how to make a small loop go through N cycles to form a large whole loop. This round of teaching practice takes 4 credit hours offline.

#### 2.3.1. Design for culture motivating.

The motivating link of the first stage of POA aims to enable students to clarify cultural knowledge gaps, enhance exploration desire, and motivate learning desire in the process of initially solving output tasks [31]. First, the teacher declares the output goal of cultural translation in this unit, which is to be able to fluently and accurately complete the English translation of this unit based on an understanding of the traditional Chinese martial arts heritage— “Tai Chi”. Students need to complete three tasks next.

Task 1: Students imagine they are introducing “Tai Chi”, the traditional Chinese martial arts event, to an international person and recommending it as a daily fitness exercise. The purpose of this task is to make students aware of their gaps in Chinese historical and cultural cultivation, triggering a strong desire for knowledge. Specific approach: students discuss in groups, and then they choose representatives to give oral presentations to the class.

Task 2: In the paragraph analysis section, specific language and cultural output barriers are proposed. Based on this, a mind map is designed to diverge relevant themes and expand content, thereby stimulating more input expectations. From the oral presentation during the driving stage, it can be seen that students have problems such as a lack of understanding of traditional culture, lack of precision in word usage, vague sentence meanings, incorrect part of speech conversion, chaotic tense, inability to handle sentence cohesion, and mistranslation of culturally loaded words. The mind maps designed by students present concepts related to “Tai Chi”, such as “intangible cultural heritage”, “theory of yin-yang and five elements”, “combination of hardness and softness”, “traditional Chinese medicine meridians”, “Shaolin boxing”, “southern style boxing”, “Wing Chun” etc. The heated discussion greatly excited students’ existing knowledge, stimulated their desire to understand relevant figures and history through relevant cultural symbols, promoted learners’ subjectivity, increased their classroom participation, and was conducive to the sustainable development of cross-cultural learning [32]. This motivating task meets the three evaluation index of this stage: communicative authenticity, cognitive challenge, and appropriateness of output goals [31].

Task 3: Students accomplish the initial translation of the “Tai Chi” paragraph (completed online). Students submit this assignment to the online learning platform.

The teaching design and learning steps of the motivating link are shown in Table 2.

**Table 2:** Design for motivating teaching and learning.

Entry	Motivating teaching steps	Motivating learning steps
1	The teacher explains the total output tasks of cultural translation related to the unit theme	Students discuss in groups, and then they choose representatives to give oral presentations to the class
2	The teacher assigns three sub-tasks to students in the enabling link	Students design mind maps present concepts related to “Tai Chi”
3	The teacher checks the output tasks completed by students and gives guidance	Students try the initial translation of the paragraph (completed online)

#### 2.3.2. Design for culture enabling.

The enabling link of the second stage of POA aims to provide multimodal material support for the cultural gap presented in the first stage, transforming input knowledge related to the theme into output knowledge [31]. Based on the analysis of learning situation, in response to the insufficient output of the cultural concept of “Tai Chi”, teachers provide reading and audio-visual materials suitable for A-level students step by step based on sufficient pre-judgment in the early stage. Then, students gradually engage in various forms of speaking, writing, and translating language output tasks that align with the unit theme. The enabling link is divided into three stages.

Step 1: Fast reading exercises (online). The teacher provides an English passage introducing “Tai Chi” and posts it on the online learning platform. The passage is an introduction to the history, characteristics, functions, and influence of “Tai Chi”, with the aim of providing students necessary background information and helping them master relevant English vocabulary and sentence structures. To increase learning effectiveness, the teacher publishes word-filling questions on the online learning platform and conduct evaluations. This task takes fast reading as input, and produces the required vocabulary and other extended topic vocabulary as oral output. The reading task is relatively simple, and students are more likely to acquire key vocabulary in small tasks, enhancing their sense of acquisition.

Step 2: Video vocabulary practice (offline). The teacher provides a 10 minute English introduction video of Chinese martial arts with English subtitles to turn the cultural symbols appearing in the previous mind map into real-life scenes. The micro output of this task for students is to capture and record the word block fragments required for Chinese English translation and the word blocks listed in the mind map during the video watching process. The difficulty of this task-based audio-visual training is moderate.

Step 3: Role play (online and offline). Before class, the teacher will cut the Chinese martial arts cultural documentary of 26-minute from CCTV-9 into four video clips with a duration of about 5 minutes, namely “Tai Chi”, “Wing Chun”, “Shaolin boxing”, and “southern style boxing”. The teacher posts the clips as a supplement to the online audio-visual training resource library of class immediately when the third step starts. The micro output of this task is a real-life simulation of the propagandists of Chinese martial arts culture, which has a certain degree of communicative authenticity. Students discuss in groups and select one of the four theme segments. Based on the reference video, they will write an English promotional message of about 120 words and make a presentation in class. They can choose to present it individually or group role-playing to complete it together. This task takes non-subtitle videos as input and converts the translation in the textbook into more difficult interpretation as output. The difficulty of the task increases, and the cultural chunks obtained in the early stages are integrated into the sentence and passage output, consolidating the new corpus.

In the next class, students are required to complete the translation of “Tai Chi” in class, and then upload their revised translations to the online learning platform. The teaching design and learning steps of the enabling link are shown in Table 3.

**Table 3:** Design for enabling teaching and steps for enabling learning.

Entry	Design for enabling Teaching	Steps for enabling learning
1	The teacher provides an English passage introducing “Tai Chi”, publishes words filling questions online and conduct evaluations	Students accomplish fast reading exercise and words filling questions online
2	The teacher provides an audio-visual material of Chinese martial arts with English subtitles	Students captures and records the word block fragments required for translation and the word blocks listed in the mind map during the video watching process
3	The teacher provides four video clips about the unit theme, posts them online and then conducts a role play task	Based on each video clip, students write an English promotional message and make a presentation in class

#### 2.3.3. Design for culture evaluating.

In the process of teaching experiment of this unit, multiple evaluations were conducted on students, including immediate and delayed evaluations, oral and written evaluations, teacher evaluations, and student-student evaluations [33]. Firstly, the author and another colleague engaged in translation teaching and research strictly follow the grading standards of CET-4/6 to grade students’ initial and revised translations of paragraphs. Secondly, the author scores the completion of online facilitation stage tasks for students and provides comments. Thirdly, after uploading the revised translation to online learning platform, students make a discussion sentence by sentence in groups to form a renew translation version, and upload it to the online learning platform before casting to the screen. Finally, students select two sets of translations for each sentence, and other groups of students identify the highlights and shortcomings of the translation. The teacher and students work together to determine the best translation version. Based on the presentation of the translated text in class and the review of the revised version, it was found that there are still two typical language errors among students: one is the consecutive translation error, and the other is the grammar problem of sentence cohesion error. In view of this, the author conducted a remedial teaching by introducing two micro lesson videos of translation skills from the online learning platform, and assigned an after-class assignment on it—revising sentences with errors (6 sentences in total). After class, the teacher continuously reviewed and provided feedback on these assignments. The teaching design and learning steps of the evaluating link are shown in Table 4 [31].

**Table 4:** Steps for evaluating teaching and steps for evaluating learning.

Entry	Steps for Evaluating Teaching	Steps for Evaluating Learning
1	The teacher scores the tasks of online motivating link for students and provides comments	Students discuss the revised translation in groups to form a new translation, and then upload it to online learning platform for screen projection
2	The teacher and students work together to determine the best translation version	Students select two sets of translations for each sentence, and other groups of students identify the highlights and shortcomings of the translation
3	After remedial teaching, the teacher reviewed and provided feedback on the after-class assignments	The teacher and students work together to determine the best translation version

## 3. Experimental results and discussion

### 3.1. Quantitative data statistics and analysis

Firstly, scores comparison of the experimental group between pre-test and post-test of cultural paragraph translation. The experiment tested the impact of POA teaching on cultural translation, but it did not involve a comparison of the initial translation and revision scores of the control group, as the revision of the control group was carried out after “checking the answers”, so the improvement in scores cannot reflect their actual translation ability. This experiment used SPSS 26.0 to conduct paired sample t-tests on the pre-test and post-test scores of the experimental group [34]. The specific data is shown in Table 5. The results showed that the mean score of the pre-test was 9.69 points (SD = 1.070), and the mean score of the post-test was 11.42 points (SD = 1.081). The difference in pre and post test scores at the 0.05 level was extremely significant. t = −11.647, p = 0.000 < 0.05.

**Table 5:** Scores comparison between pre-test and post-test of “Tai Chi” translation in the experimental group.

Test	Number of cases	Mean	Standard Deviation	t value	p value
Pre-test	31	9.69	1.070	−11.647	0.000
Post-test	31	11.42	1.081		

Under the guidance of POA, the redesign and implementation of cultural paragraph translation teaching has significantly improved students’ translation quality and cultural translation ability. These improvements are specifically manifested in: (1) The revised translation is more fluent; (2) Fewer grammar errors in revised translations; (3) The words used in the revised translation are more appropriate; (4) More accurate expressions related to Chinese culture. In addition, the average score of the 6 post-class sentence error correction related to continuous verb translation and sentence cohesion in remedial teaching is 26.7 out of 30, indicating that students’ awareness of part of speech has been improved and their understanding of grammatical structures has become clearer.

Secondly, translation scores comparison in the final exam translation between the control group and the experimental group. The translation in the final exam is a paragraph translation with the theme of “Chinese Filial Piety”. After the test paper is collected, two teachers from the same group grade the translation parts of the two groups according to the grading standards of CET-4 and CET-6. The author used SPSS 26.0 software to compare the translation scores between the control group and the experimental group [35]. The independent sample t-test results showed that the average score of the control group was 9.98 (SD = 1.076), while the average score of the experimental group was 11.92 (SD = 0.932). The difference between the two groups of students was significant at the 0.05 level, with t = −7.570 and p = 0.000 < 0.05 (see Table 6). Data analysis shows that after a semester of translation training under the guidance of POA, the experimental group’s translation ability has been improved compared to the control group, which indicates that POA can effectively improve students’ translation quality and enhance their translation ability. The specific data is shown in Table 6.

**Table 6:** Scores comparison in the final exam between the control group and experimental group.

Group	Number of cases	Mean	Standard Deviation	t value	p value
Control group	31	9.98	1.076	−7.570	0.000
Experimental group	31	11.92	0.932		

### 3.2. Qualitative data statistics and analysis

After the one-semester experiment on the college English cultural translation section under the guidance of POA, the author collected some supplementary data in the form of questionnaires and interviews to understand students’ acceptance and satisfaction with POA, and further verify the effectiveness of the teaching method [36].

#### 3.2.1. Survey for students.

**Fig 3:**
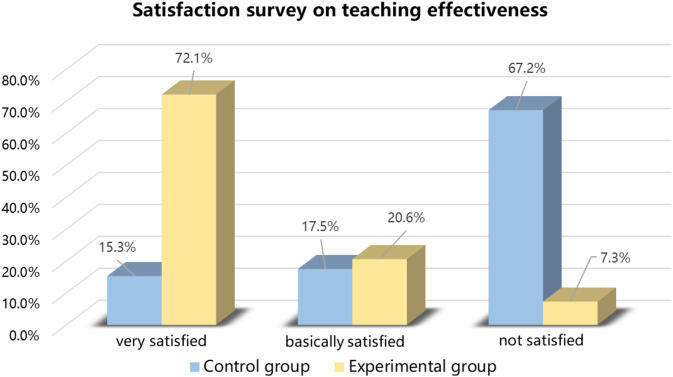
Satisfaction comparison between control group and experimental group on teaching effectiveness.

The author used the Questionnaire Star app to conduct a questionnaire on teaching cultural paragraph translation, with a total of twelve questions, of which ten were objective questions, and the last two were objective questions. At the end of the semester, electronic questionnaires will be sent to the experimental and control group students to fill out. The satisfaction comparison between POA cultural translation teaching and traditional translation teaching is shown in Fig 3.

At the same time, the author randomly selected ten students from the control group and ten students from the experimental group for semi-structured online interviews. Students of both groups believed that learning cultural translation is “quite important”. They all considered that “insufficient vocabulary” and “difficulty in achieving authentic English expression” are the most difficult problems when they encountered in translation. Seven students in the experimental group expressed that the online and offline teaching method based on POA is “novel” and “it aroused translation interest”. Six students in the control group thought that the translation class was “a bit boring”, paragraph translation was “a bit difficult”, and “they couldn’t help searching for translation answer” when doing exercises. The students in the experimental group all believed that the enabling process and remedial teachings had “great inspiration” and “targeted effects” for cultural translation learning. Through the POA teaching method, they can “enrich the vocabulary”, “words usage has become more appropriate”, “grammar errors have been reduced”, and “sentence writing has become more fluent”. What’s more, “the expression ability of Chinese culture has been obviously improved”. Eight students in the experimental group believed that the teaching experiment had “satisfactory results” and “greatly benefited”. But two of the students believed that “the assigned too much learning tasks and the pressed time are something hard for them, even the teaching content cannot be fully absorbed”. However, nine students in the control group were not satisfied with the current translation teaching. They hoped that the teacher can explore translation teaching methods, supplement translation skills and cultural knowledge, and English classroom are more funny so as to truly help students improve their cultural translation ability.

Overall, the students in the experimental group held a positive and affirmative attitude towards the application of POA in the teaching experiment of cultural translation [37]. They believed that this teaching method is novel, unique and sustainable, which helped to enhance translation interest and confidence, expand their English vocabulary, improve the accuracy of vocabulary usage, and standardize grammar usage. More importantly, college English translation course guided by POA has deepened students’ understanding of Chinese history and enhance their sense of cultural identity and pride, which has a significant promoting effect on the improvement of the English expression ability of the mother tongue culture and the external communication ability of Chinese culture, conforming to the goal of sustainable development of education.

#### 3.2.2. Reflection for teachers.

The significant improvement in the translation scores, final translation scores, and after-class homework quality of the experimental group students, as well as their active cooperation and recognition of this teaching experiment, greatly enhanced the author’s confidence in applying POA for online and offline mixed teaching reform of cultural paragraph translation. The application and implementation of POA in the classroom have proposed a new paradigm for A-level students in the input and output patterns of Chinese culture. Teachers need to pay attention to optimizing teaching design, arranging teaching processes, applying teaching strategies, organizing classroom activities, and enhancing the application of modern educational technology in the application of this teaching concept, in order to promote the development of blended online and offline teaching. But at the same time, the author also deeply realizes that there are many challenges faced in the design and implementation of POA teaching [38].

Teachers need to accurately assess the language proficiency and cognitive abilities of A-level students, scientifically set the difficulty of enabling output tasks, and select input language and cultural materials that pose certain challenges to promote the completion of small output goals, thereby achieving finial output goals. For teachers, the most challenging part lies in accurately estimating students’ cognitive and language abilities, collecting and selecting the most suitable multimodal learning resources, and then setting output subtasks that match the difficulty and learning situation, effectively helping students achieve teaching objectives. On the one hand, teachers need to pay attention to the accumulation of language materials and enrich teaching materials in their daily lives. On the other hand, a teaching team can be organized to jointly build a teaching resource library and collectively discuss lesson preparation. The English teaching team of our college has accumulated and organized a large amount of Chinese and English cultural materials through years of teaching practice, and has initially established a multimodal resource library for cross-cultural teaching in our school, covering more than twenty themes matched the first to fourth volumes of *New Horizon College English*. The resources include TED speeches, film and television cultural clips, Chinese and English documentaries, creation of WeChat official accounts of Chinese traditional culture, English talk show programs, photos and music database, and so on. With the continuous improvement of the resource library, teachers, as intermediaries, have more diverse choices for the selection of multimodal input data in the enabling process, ensuring the effective completion of student output tasks on the basis of assessing the appropriateness of materials.

#### 3.2.3. Discussion.

The effectiveness of applying POA theory to Chinese cultural translation teaching is determined by the interaction of three stages: motivating, enabling and evaluating. In the motivating phase, students clarify their learning goals, be aware of their shortcomings, thereby improving their learning motivation. In the enabling stage, the teachers play a leading role by providing sufficient input materials to help students complete output tasks and improve language skills. In the evaluating process, the quality of students’ output is further improved. At the same time, the results of evaluating help teachers realize the teaching effect and thus complete remedial teaching strategies.

Most previous studies in China only had simple samples, and the “output task” was only approached from the perspective of teachers, ignoring individual differences among students. Meanwhile, there is a lack of research on the process-oriented approach to translation teaching, as well as empirical studies on the motivating process, which is not operational. This study is a systematic mixed teaching experiment for one-semester on the effectiveness of POA for A-level English learners, which is based on the process-oriented research of POA teaching design, strengthening the process-oriented design of student output in the “motivating, enabling and evaluating” processes, and refining the evaluation indicators.

## 4. Conclusion

Unlike traditional task-based teaching, blended cultural translation teaching based on POA theory can guide students to discover problems and solve problems through continuous learning, which is more conducive to stimulating students’ initiative and sustainable development. The teaching model of language-driven culture enhances the key pragmatic abilities and Chinese cultural output abilities of A-level students in college English hierarchical teaching. This model not only has important implications for the translation guidance of cultural paragraphs in CET-4 and CET-6 but also benefits college students in inheriting and developing China’s excellent traditional culture, establishing confidence for national culture. How to effectively teach college English translation based on POA is a major challenge for frontline teachers. It is implicated that the proportion and intensity of culture translation should be increased in college English teaching. To better implement the teaching of this theory, teachers should further optimize teaching design in the teaching process, accurately set driving tasks based on the cultural translation section, enrich formative task resources, and diversify evaluation methods. At the same time, college English teaching should attach importance to more textbook development guided by POA, as well as regular online or offline training and Q&A for frontline teachers, providing more accurate and comprehensive assistance for sustainable development of teaching and learning of college English translation.
